# On the AIC-based model reduction for the general Holzapfel–Ogden myocardial constitutive law

**DOI:** 10.1007/s10237-019-01140-6

**Published:** 2019-04-03

**Authors:** Debao Guan, Faizan Ahmad, Peter Theobald, Shwe Soe, Xiaoyu Luo, Hao Gao

**Affiliations:** 1 School of Mathematics and Statistics, University of Glasgow, Glasgow, UK; 2 School of Engineering, Cardiff University, Cardiff, UK

**Keywords:** Akaike information criterion (AIC), Holzapfel–Ogden (HO) constitutive law, Reduced HO models, Simple shear tests, Uniaxial tests, Biaxial tests, Myocardial mechanical tests

## Abstract

Constitutive laws that describe the mechanical responses of cardiac tissue under loading hold the key to accurately model the biomechanical behaviour of the heart. There have been ample choices of phenomenological constitutive laws derived from experiments, some of which are quite sophisticated and include effects of microscopic fibre structures of the myocardium. A typical example is the strain-invariant-based Holzapfel–Ogden 2009 model that is excellently fitted to simple shear tests. It has been widely used and regarded as the state-of-the-art constitutive law for myocardium. However, there has been no analysis to show if it has both adequate descriptive and predictive capabilities for other tissue tests of myocardium. Indeed, such an analysis is important for any constitutive laws for clinically useful computational simulations. In this work, we perform such an analysis using combinations of tissue tests, uniaxial tension, biaxial tension and simple shear from three different sets of myocardial tissue studies. Starting from the general 14-parameter myocardial constitutive law developed by Holzapfel and Ogden, denoted as the general HO model, we show that this model has good descriptive and predictive capabilities for all the experimental tests. However, to reliably determine all 14 parameters of the model from experiments remains a great challenge. Our aim is to reduce the constitutive law using Akaike information criterion, to maintain its mechanical integrity whilst achieving minimal computational cost. A competent constitutive law should have descriptive and predictive capabilities for different tissue tests. By competent, we mean the model has least terms but is still able to describe and predict experimental data. We also investigate the optimal combinations of tissue tests for a given constitutive model. For example, our results show that using one of the reduced HO models, one may need just one shear response (along normal-fibre direction) and one biaxial stretch (ratio of 1 mean fibre : 1 cross-fibre) to satisfactorily describe Sommer et al. human myocardial mechanical properties. Our study suggests that single-state tests (i.e. simple shear or stretching only) are insufficient to determine the myocardium responses. We also found it is important to consider transmural fibre rotations within each myocardial sample of tests during the fitting process. This is done by excluding un-stretched fibres using an “effective fibre ratio”, which depends on the sample size, shape, local myofibre architecture and loading conditions. We conclude that a competent myocardium material model can be obtained from the general HO model using AIC analysis and a suitable combination of tissue tests.

## Introduction

Cardiac diseases remain a major public healthy burden, especially the adverse remodelling of cardiac function after acute myocardial infarction. Studies have demonstrated that stress/strain in myocardium can have great effects on pathological processes such as hypertrophy and myocardial infarction (Zile et al. 2004; Costa et al. 2001; Mangion et al. 2017). Accurate prediction of myocardial stress relies on the choice of constitutive laws. Determining the constitutive laws and their parameters from limited experimental data, however, remains a great challenge for the cardiac modelling community.

In general, biological tissue, including myocardium, mainly consists of proteins such as collagen, elastin and ground substance. Published in vitro/ex vivo experimental tests of the mechanical behaviour of human myocardium (Pinto and Fung 1973) have shown strong anisotropy and transmural variations. Similar conclusions have also been reported by other studies, with Langdon et al. (1999) investigating the effect of biaxial constraint caused by glutaraldehyde cross-linking on the equal-biaxial mechanical properties of bovine pericardium. Dokos et al. (2002) examined the shear properties of passive ventricular myocardium through six modes of simple shear tests on samples from porcine hearts, reporting that simple shear responses are highly nonlinear along the micro-structural axes of the tissue. Later, Sommer et al. (2015b) determined biaxial extension and triaxial shear properties, characterizing the underlying micro-structure of the passive human ventricular myocardium. Results showed it is a nonlinear, anisotropic (orthotropic), viscoelastic and history-dependent soft biological material that undergoes large deformations. Very recently, Ahmad et al. (2018) studied biomechanical properties of neonatal porcine cardiac tissue by using uniaxial tensile, biaxial tensile and simple shear loading modes with samples from the anterior and posterior walls of the right and left ventricles. The compressibility of myocardial tissue is quantified by McEvoy et al. (2018) using a joint experimental-computational approach, investigating volumetric changes in excised porcine myocardium tissue under both tensile and confined compression loading conditions.

Over the years, a number of models have been developed to describe myocardial mechanical properties, ranging from linear elastic to hyperelastic, from isotropic to anisotropic, and from phenomenological to microstructurally based constitutive laws (Holzapfel and Ogden 2009). Nowadays, it is a common practice to characterize myocardium as an anisotropic, hyperelastic material. One approach employs the angular integration of each collagen fibre’s contribution following a distribution map. Lanir (1983) developed a general multi-axial theory for the constitutive relations in fibrous connective tissues on the basis of micro-structural and thermodynamic considerations. Sacks et al. (2016) developed a rigorous full structural model (i.e. explicitly incorporating various features of the collagen fibre architecture) for exogenously cross-linked soft tissues, which made an extension to the collagenous structural constitutive model, meaning the uncross-linked collagen fibre responses could be mapped to the cross-linked configuration. Based on Sack’s study, Avazmohammadi et al. (2017b) proposed a fibre-level constitutive model for the passive mechanical behaviour of the right ventricular free wall, which explicitly separated the mechanical contributions of myocytes and collagen fibre ensembles, whilst accounting for their mechanical interactions.

Another widely used approach employs strain components directly or strain invariants when developing such constitutive laws. For instance, Guccione et al. (1991) used a transverse isotropic exponential Fung-type hyperelastic material model to characterize the equatorial region of the canine left ventricle, in which the strain energy function consists of six strain components. Soon afterwards, LeGrice et al. (1995) found that the micro-structure of myocardium was a composite of discrete fibre layers, which suggested an orthotropic mechanical response according to a local orthotropic material axes: the fibre direction ${\mathbf {f}}$, the sheet direction ${\mathbf {s}}$ and the sheet–normal ${\mathbf {n}}$. The transversely isotropic Fung-type relation was then extended to account for the orthotropy described by Costa et al. (2001). There are many constitutive laws that use strain-invariant-based orthotropic or transversely isotropic constitutive laws to characterize passive myocardial tissue, which were recently reviewed in Holzapfel and Ogden (2009). Based on the simple shear data from Dokos et al. (2002), Holzapfel and Ogden proposed a simplified formulation (HO2009) derived from a more general strain-invariant-based material model (the general HO model) (Holzapfel and Ogden 2009). The HO2009 model has one term related to the matrix responses, two terms related to the stress responses along ${\mathbf{f}}$ and ${\mathbf{s}}$, and a final term for interaction between ${\mathbf{f}}$ and ${\mathbf{s}}$.

The HO2009 model and its variation have been widely used in the cardiac modelling community such as the LivingHeart Project (Baillargeon et al. 2014). Göktepe et al. (2011) developed a general constitutive and algorithmic approach to the computational modelling of passive myocardium using the HO2009 model, which is embedded in a nonlinear finite element method. Wang et al. (2013) studied the fibre orientation on left ventricular diastolic mechanics using the HO2009 model and further extended it to include residual stresses (Wang et al. 2014). Gao et al. (2017) implemented the HO2009 model into an immersed boundary framework combined with finite element to study left ventricle (LV) biomechanics both in diastole and systole. Simplified forms of the HO2009 model were also used by Asner et al. (2016) with personalized ventricular dynamics derived from in vivo data. General structural tensors accounting for collagen fibre dispersion were introduced by Eriksson et al. (2013), followed by the recent extension of Melnik et al. (2018) to account for fibre dispersion in the coupling term between the fibre and sheet directions. Inverse estimation of unknown parameters in the HO2009 model from in vivo data was first investigated by Gao et al. (2015), and later by Nikou et al. (2015), and by Palit et al. (2018). The HO2009 model has also been applied to simulate various heart diseases such as myocardial infarction (Gao et al. 2017; Baillargeon et al. 2014).

No study has previously investigated the descriptive and predictive capability of HO-type strain energy functions. A competent constitutive law should be able to describe as many deformation modes (uniaxial, biaxial, simple shear, etc.) as possible in qualitative point and then from quantitative point, with acceptable errors of simulation with respect to the experimental data (Destrade et al. 2017), and have the fewest terms. Mechanical properties of myocardium are traditionally measured by a single series of either uniaxial (Pinto and Fung 1973), biaxial tests (Demer and Yin 1983) or simple shear deformations (Dokos et al. 2002), despite it being demonstrated that combined biaxial data (with different loading protocols) and simple shear data (with various loading directions) are required to adequately capture the tissue’s direction-dependent nonlinear response (Holzapfel and Ogden 2009). For example, Holzapfel and Ogden (2009), and Schmid et al. (2009) both only used simple shear data of Dokos et al. (2002) to demonstrate the good descriptive capability of selected constitutive laws. Only recently Sommer et al. (2015b) have performed both biaxial and shear tests on similar human myocardial samples, whilst Ahmad et al. (2018) reported their experiments on neonatal porcine myocardium samples with uniaxial, biaxial and shear tests. An unanswered question is whether a selected material model, such as the HO2009 model, can adequately fit to different types of mechanical tests.

A competent constitutive law should also be able to predict stress responses from different deformation modes. A constitutive law with parameters derived from simple shear test data can, for example, be used to accurately predict the biaxial test data. This predictive capability is critical for achieving accurate cardiac modelling, where the deformation states can differ significantly from the original experimental data. Some studies describe the predictive capability of constitutive laws for arterial tissues, but rarely consider myocardium. For example, Hollander et al. (2011) compared the descriptive and predictive powers of a Fung-type exponential phenomenological model, a strain-invariant-based partial structure model and a structural model based on angular integration, by characterizing coronary arterial media. They found that different test protocols (extension, inflation, and twist) are necessary to reliably predict mechanical response. Polzer et al. (2015) studied the ability of a material model to predict the biaxial response of porcine aortic tissue with a predefined collagen structure. Schroeder et al. (2018), recently, showed that the Holzapfel–Gasser–Ogden model with generalized structure tensors (Gasser et al. 2006) cannot predict the biaxial arterial wall behaviour when determined from only uniaxial tests, whilst the four-fibre-family constitutive law is the most robust when predicting uniaxial or biaxial behaviour of porcine aortic tissue.

This study first considers the descriptive capabilities of the general and specific HO models proposed in Holzapfel and Ogden (2009), using Dokos et al. simple shear data of porcine myocardium (Dokos et al. 2002), Sommer et al. biaxial and simple shear data of human myocardium (Sommer et al. 2015b), and Ahmad et al. uniaxial, biaxial and simple shear data of neonatal porcine myocardium (Ahmad et al. 2018). Secondly, the Akaike information criterion (AIC) (Schmid et al. 2006; Ten Eyck and Cavanaugh 2018; Avazmohammadi et al. 2017a) is used to analyse the goodness of fit of the general HO model to the experimental data, with AIC values determined when excluding different strain invariants. Based on the AIC analysis, reduced HO models for different experimental studies are then proposed by excluding those strain invariants with little contribution to the overall goodness of fit. Finally, we use predictive capability of the reduced HO models to find the optimal combination of experiments for each species of tissues that uses minimal mechanical tests.

## Method

### Selected myocardial experiments

In this study, the experimental data are taken from three ex vivo myocardial biomechanical studies: Dokos et al. (2002) investigating porcine myocardium; Sommer et al. (2015b) investigating human myocardium; and Ahmad et al. (2018) investigating neonatal porcine myocardium. These are briefly summarized below. For details, please refer to the original papers.

**Fig. 1:**
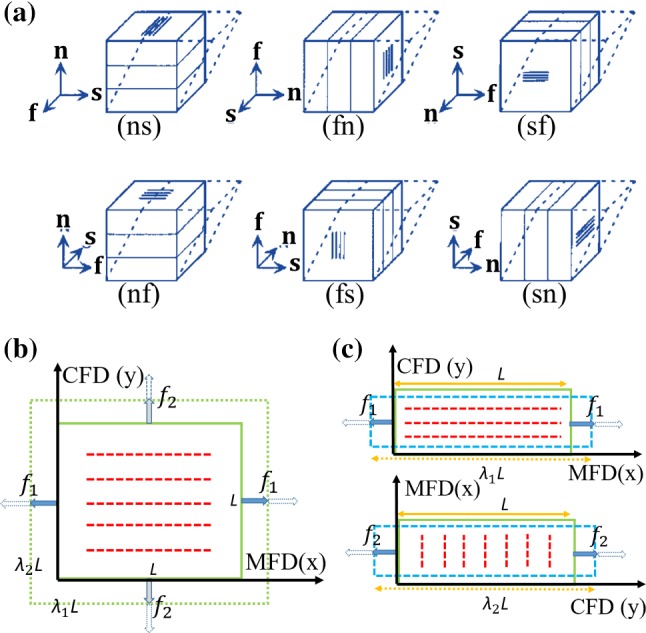
**a** A sketch of all six possible shear modes, ${\mathbf{f}}$, ${\mathbf{s}}$, and ${\mathbf{n}}$ denote the fibre, sheet, and normal direction, respectively. (*ij*) refers to shear in the *j* direction within the *ij* plane, where $i\ne j\in {\{\text {f, s, n}\}}$. **b** A sample with fibres (red dash lines), which is stretched along the two orthogonal directions (MFD and CFD) in fibre-normal plane during a biaxial test; **c** uniaxial tension tests along the MFD and CFD; $f_1$ and $f_2$ are the loading force along the MFD and CFD. *L* is the initial length of specimen, and $\lambda _1$ and $\lambda _2$ are stretch ratios

- Dokos et al. (2002) published shear data of passive myocardium from porcine hearts with six different shear modes, shown in Fig. 1a where (*ij*) is used to refer to shear in the *j* direction within the *ij* plane, where $i\ne j\in {\{\text {f, s, n}\}}$. Myocardial samples were cut from adjacent regions in the left lateral ventricular mid-wall with a size of $\sim \,3\times 3\times 3$ mm.
- Sommer et al. (2015b) performed similar six shear-mode experiments, with samples from human hearts (size: $\sim \,4\times 4\times 4$ mm). They also performed biaxial testing with different stretch ratios (1:1, 1:0.75, 1:0.5, 0.75:1, 0.5:1) along the mean fibre direction (MFD) and the cross-fibre direction (CFD) (Fig 1b). MFD is the average angle of the dominant orientation of collagen fibres on the upper and lower surfaces of each sample (Sommer et al. 2015a), with CFD perpendicular to MFD. Square specimens with dimensions $\sim \,25\times 25\times 2.3$ mm were used in biaxial tests, with tension applied along the MFD and CFD. They recorded the collagen fibre rotation within samples, which was   $14.8\pm \,6.9^\circ$ per mm depth in the transmural direction.
- Ahmad et al. (2018) performed uniaxial (Fig. 1c), biaxial and simple shear experiments on myocardial samples from neonatal porcine left and right ventricular free walls. Sample dimensions were $\sim \,15\times 5\times 3$ mm for uniaxial tests, $\sim \,15\times 15\times 3$ mm for biaxial tests and $\sim \,3\times 3\times 3$ mm for simple shear tests. Shearing was only performed in the sheet–fibre and sheet–normal planes, whilst the MFD was determined based on the external surface texture and not the average angle of the dominant orientation of collagen fibres as in Sommer et al. (2015a).

In the following, we refer these three sets of experiments as Dokos’s data, Sommers’s data, and Ahmad’s data. Let 1, 2, 3 represent the components in MFD, CFD and sheet (transmural) directions and assume that the test sample is incompressible. To make use of the experiments, it is convenient to use the first Piola–Kirchhoff (P–K) stress ${\mathbf{P}}$, which is related to the applied force components $f_{ij}$ in the tests and Cauchy stress tensor ${{\varvec{\sigma }}}$ as

1 $$\begin{aligned} {\mathbf{P}}=\left[ \begin{array}{lll} P_{11} &\quad P_{12} &\quad P_{13} \\ P_{21} &\quad P_{22} &\quad P_{23} \\ P_{31} &\quad P_{32} &\quad P_{33} \end{array}\right] =\left[ \begin{array}{lll} \frac{f_{11}}{A_1} &\quad \frac{f_{12}}{A_2} &\quad \frac{f_{13}}{A_3} \\ \frac{f_{21}}{A_1} &\quad \frac{f_{22}}{A_2} &\quad \frac{f_{23}}{A_3} \\ \frac{f_{31}}{A_1} &\quad \frac{f_{32}}{A_2} &\quad \frac{f_{33}}{A_3} \end{array}\right] , \quad {{{\varvec{\sigma }}}}=\frac{1}{\det ({\mathbf{F}})}{\mathbf{P}}{\mathbf{F}}^{T}, \end{aligned}$$

where ${\mathbf{F}}$ is deformation gradient tensor.

**Fig. 2:**
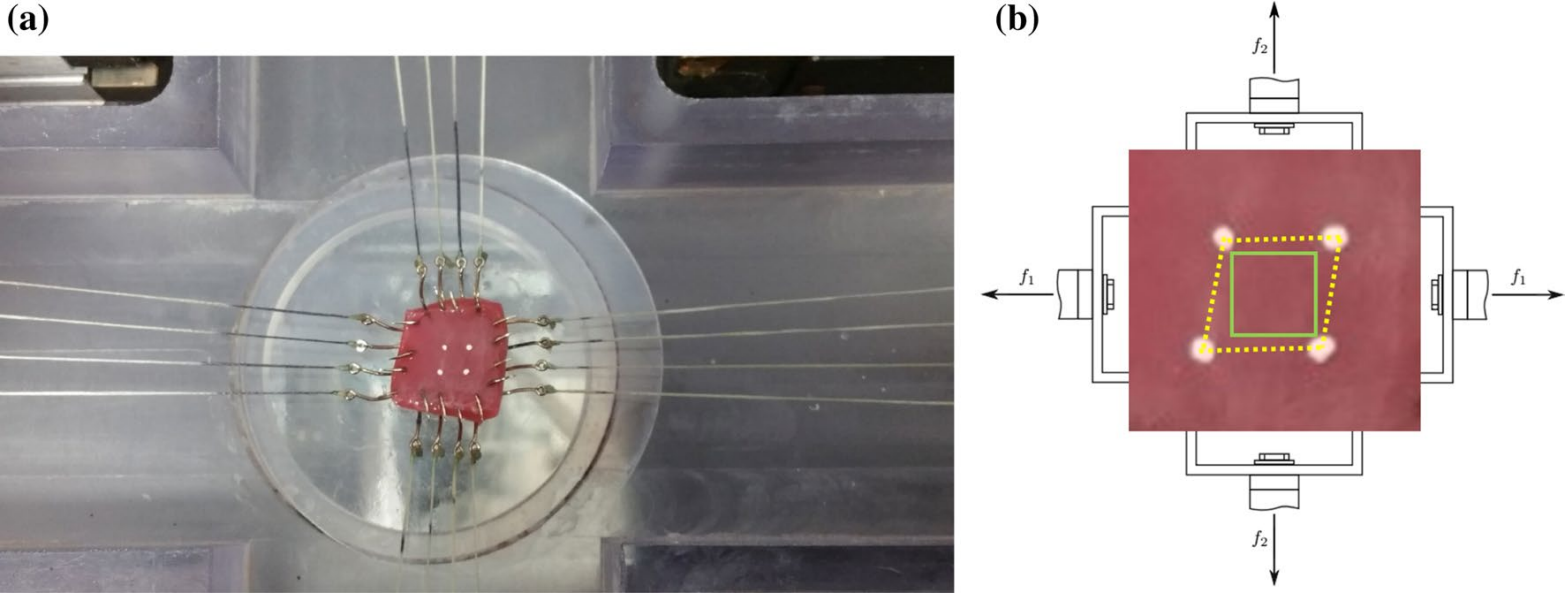
The recorded image in a biaxial tensile specimen in Ahmad et al. (2018) (**a**). The four white markers in the centre of the experimental sample in **a** are also shown in **b**, in which the solid rectangle represents the initial shape; and the deformed shape is shown in dashed lines. $f_1$ and $f_2$ are the loading forces in MFD and CFD

- **Uniaxial tests**
  For uniaxial stretch experiments along MFD, we have
  2 $$\begin{aligned} {\mathbf{F}}=\left[ \begin{matrix}\lambda _1 &\quad 0 &\quad 0 \\ 0 &\quad \frac{1}{\sqrt{\lambda _1}} &\quad 0 \\ 0 &\quad 0 &\quad \frac{1}{\sqrt{\lambda _1}} \end{matrix}\right] \,\,\,\text {and}\,\quad {\sigma }_{11}=\lambda _1\frac{f_1}{A_1}= \lambda _1 P_{11}, \end{aligned}$$
  in which $\lambda _1$ is the stretch ratio, $f_1$ is the applied force along MFD direction, and in this case $f_1=f_{11}$, ${\sigma }_{11}$ is the Cauchy stress component, and $A_1$ is the reference cross-sectional area perpendicular to MFD. Similarly, for uniaxial stretch along CFD
  3 $$\begin{aligned} {\mathbf{F}}=\left[ \begin{array}{ccc}\frac{1}{\sqrt{\lambda _2}} &\quad 0 &\quad 0 \\ 0 &\quad \lambda _2 &\quad 0 \\ 0 &\quad 0 &\quad \frac{1}{\sqrt{\lambda _2}} \end{array}\right] \,\,\,\text {and}\,\quad {\sigma }_{22}=\lambda _2\frac{f_2}{A_2}=\lambda _2 P_{22}, \end{aligned}$$
  where the applied force $f_2=f_{22}$.
- **Biaxial tests**
  For the shear-free biaxial test along MFD and CFD, since $A_1 = A_2 = A$, then
  4 $$\begin{aligned} {\mathbf{F}}=\left[ \begin{matrix} \lambda _1 &\quad 0 &\quad 0 \\ 0 &\quad \lambda _2 &\quad 0 \\ 0 &\quad 0 &\quad \frac{1}{\lambda _1\,\lambda _2} \end{matrix}\right] \, \text {and} \, {\sigma }_{11}=\lambda _1\frac{f_1}{A}=\lambda _1 P_{11}, \, {\sigma }_{22}=\lambda _2\frac{f_2}{A}=\lambda _2 P_{22}. \end{aligned}$$
  Again, in this case, we have $f_1=f_{11}$, $f_2=f_{22}$.
  If shear exists in the biaxial test as in Fig. 2, $\gamma _{12}\ne 0 \,\text {and}\,\gamma _{21}\ne 0$, then
  5 $$\begin{aligned} {\mathbf{F}}=\left[ \begin{matrix} \lambda _1 &\quad \gamma _{12} &\quad 0 \\ \gamma _{21} &\quad \lambda _2 &\quad 0 \\ 0 &\quad 0 &\quad \frac{1}{\lambda _1\,\lambda _2-\gamma _{12}\,\gamma _{21}} \end{matrix}\right] \, \text {and} \, {\sigma }_{11}=\lambda _1\,P_{11}+\gamma _{12}\,P_{12},\, {\sigma }_{22}=\lambda _2\,P_{22}+\gamma _{21}\,P_{21}. \end{aligned}$$
  Because the measured force is the sum of forces along the directions 1 and 2, there is no force applied in the third direction, $f_1$ and $f_2$ satisfy the following equations (Sommer et al. 2015a)
  6 $$\begin{aligned} f_1=f_{11}+f_{12},\,\,\text {and}\,\,f_2=f_{21}+f_{22}. \end{aligned}$$
  Finally, the relationship between the first P–K stress components and the applied forces is
  7 $$\begin{aligned} \begin{aligned}&P_{11}+P_{12}=({{\varvec{\sigma }}}{\mathbf{F}}^{-\text {T}})_{11}+({{\varvec{\sigma }}} {\mathbf{F}}^{-\text {T}})_{12} = \frac{f_1}{A} ,\\&P_{21}+P_{22}=({{\varvec{\sigma }}}{\mathbf{F}}^{-\text {T}})_{21}+({{\varvec{\sigma }}} {\mathbf{F}}^{-\text {T}})_{22} = \frac{f_2}{A}. \end{aligned} \end{aligned}$$
  Note when shear is present, $\sigma _{11}\ne \lambda f_1/A$ and $\sigma _{22}\ne \lambda f_2/A$. Therefore, we need to determine the P–K stress components from experiments using (7), and then recover the Cauchy stress components from (1).
  We further assume the shear increases linearly with stretch, that is
  8 $$\begin{aligned} \gamma _{12}=k_1\frac{\lambda _1-1}{\lambda _1^{\max }-1},\quad \text {and} \quad \gamma _{21}=k_2\frac{\lambda _2-1}{\lambda _2^{\max }-1}, \end{aligned}$$
  where $k_1$ and $k_2$ are the maximum values of $\gamma _{12}$ and $\gamma _{21}$.
- **Simple shear tests**
  For the simple shear tests, shown in Fig. 1a, we have
  9 $$\begin{aligned} \text {(ns):} \quad {\mathbf{F}}=\left[ \begin{matrix} 1 &\quad 0&\quad 0 \\ 0 &\quad 1 &\quad 0 \\ 0 &\quad \gamma _{32} &\quad 1 \end{matrix}\right] \quad \text {(fn):} \quad {\mathbf{F}}=\left[ \begin{matrix} 1 &\quad 0 &\quad 0 \\ \gamma _{21} &\quad 1 &\quad 0 \\ 0 &\quad 0 &\quad 1 \end{matrix}\right] \quad \text {(sf):} \quad {\mathbf{F}}=\left[ \begin{matrix} 1 &\quad 0 &\quad \gamma _{13} \\ 0 &\quad 1 &\quad 0 \\ 0 &\quad 0 &\quad 1 \end{matrix}\right] \nonumber \\ \text {(nf):} \quad {\mathbf{F}}=\left[ \begin{matrix} 1 &\quad \gamma _{12} &\quad 0 \\ 0 &\quad 1 &\quad 0 \\ 0 &\quad 0 &\quad 1 \end{matrix}\right] \quad \text {(fs):} \quad {\mathbf{F}}=\left[ \begin{matrix} 1 &\quad 0 &\quad 0 \\ 0 &\quad 1 &\quad 0 \\ \gamma _{31} &\quad 0 &\quad 1 \end{matrix}\right] \quad \text {(sn):} \quad {\mathbf{F}}=\left[ \begin{matrix} 1 &\quad 0 &\quad 0 \\ 0 &\quad 1 &\quad \gamma _{23} \\ 0 &\quad 0 &\quad 1 \end{matrix}\right] \end{aligned}$$
  and in this case, the stress components are determined from
  10 $$\begin{aligned} \sigma _{ij}={P}_{ij} =\frac{f_{ij}}{A},\,\,\,\,i\ne \,j\in \{1,2,3\}. \end{aligned}$$

### The general HO model

To characterize the mechanical behaviours of myocardium, the general HO strain energy function proposed by Holzapfel and Ogden (Holzapfel and Ogden 2009) is employed, which is

11 $$\begin{aligned} \begin{aligned} \varPsi&=\frac{a}{2b}\exp [b(I_1-3)]+\sum _{i=\text {f,s,n}}\,\frac{a_i}{2b_i} \{\exp [b_i( \max (I_{4i}, 1)-1)^2]-1\} \\&\quad +\sum _{ij=\text {fs,fn,sn}}\,\frac{a_{ij}}{2b_{ij}}[\exp (b_{{ij}}I_{8ij}^2)-1], \end{aligned} \end{aligned}$$

where $a, b, a_i, b_i, a_{ij}, b_{ij}$ are the 14 material constants, $I_1 = \text {trace}({\mathbf{F}}^T {\mathbf{F}})$, representing the overall squared stretch, $I_\text {4f}$, $I_\text {4s}$ and $I_\text {4n}$ are squared stretches along each direction,

$$\begin{aligned} I_\text {4f} = {\mathbf{f}}_0 \cdot ({\mathbf{F}}^T{\mathbf{F}}\, \mathbf{f}_0), \quad I_\text {4s} = \mathbf{s}_0 \cdot (\mathbf{F}^T\mathbf{F}\, \mathbf{s}_0), \quad I_\text {4n} = \mathbf{n}_0 \cdot (\mathbf{F}^T\mathbf{F}\, \mathbf{n}_0), \end{aligned}$$

in which $\mathbf{f}_0, \mathbf{s}_0, \mathbf{n}_0$ are the initial fibre, sheet and normal directions. The $\max ( )$ in (11) will ensure the collagen fibres can only bear the load when stretched but not in compression. $I_\text {8fs}$, $I_\text {8fn}$ and $I_\text {8sn}$ are invariants representing the coupling between two different directions,

$$\begin{aligned} I_\text {8fs} = \mathbf{f}_0 \cdot (\mathbf{F}^T\mathbf{F}\, \mathbf{s}_0), \quad I_\text {8fn} = \mathbf{f}_0 \cdot (\mathbf{F}^T\mathbf{F}\, \mathbf{n}_0), \quad I_\text {8sn} = \mathbf{s}_0 \cdot (\mathbf{F}^T\mathbf{F}\, \mathbf{n}_0). \end{aligned}$$

### Effective fibre contribution

**Fig. 3:**
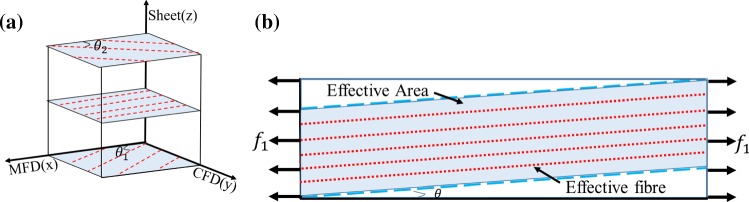
**a** The fibre direction varies along the thickness of myocardium. **b** The effective area (blue) when the fibre direction is $\theta$ under uniaxial loading in the MFD test. The collagen fibres (red dot line) within the region enclosed by the two blue dashed lines are defined as effective fibres that are stretched both sides. The effective fibre ratio is defined by rectangle area dividing blue effective area

The rotation of collagen fibres from epicardium to endocardium plays a significant role in the myocardial mechanical response. Thus, it is necessary to consider fibre rotation in tested samples when fitting constitutive laws to experimental data. We further assume collagen fibres (along with myocytes) only lie in the $\mathbf{f}-\mathbf{n}$ plane. Considering a myocardial sample with linearly rotated fibres from $\theta _1$ to $\theta _2$ as shown in Fig. 3a, the local fibre angle related to the MFD with a depth of *h* is

12 $$\begin{aligned} \theta (h)=\frac{\theta _2-\theta _1}{H_0} \,h+\theta _1, \end{aligned}$$

where $H_0$ is the total thickness of the sample, and the local $\mathbf{f}$–$\mathbf{n}$–$\mathbf{s}$ system is

$$\begin{aligned} \mathbf{f}=(\cos {\theta }, \sin {\theta }, 0), \quad \mathbf{n}=(-\sin {\theta }, \cos {\theta }, 0) \quad \text {and} \quad \mathbf{s}=(0, 0, 1). \end{aligned}$$

Because collagen fibres can only bear the load when stretched, factors depending on fibre angle ($\theta$) are introduced in the general HO law for $I_\text {4f}$, $I_\text {4s}$ and $I_\text {4n}$ which measure the squared stretches of different fibre families, but not for $I_\text {8fs}$, $I_\text {8fn}$ and $I_\text {8sn}$ which are dependent on the angels between different directions. Therefore,

13 $$\begin{aligned} \varPsi (\theta )= & \psi _1+\alpha _{\text {4f}}(\theta )\psi _{4f}\nonumber \\&+\alpha _{\text {4s}}(\theta )\psi _{4s}+\alpha _{\text {4n}}(\theta )\psi _{4n}+\psi _\text {8fs}+\psi _\text {8fn}+\psi _\text {8sn}, \end{aligned}$$

in which $\psi _i$ is the strain energy term associated with the invariant of $I_i$. $\alpha _{\text {4f}}(\theta )$, $\alpha _{\text {4s}}(\theta )$ and $\alpha _{\text {4n}}(\theta )$ values will depend on the experimental loading conditions and the fibre structure of tested samples. For example, in an uniaxial test along the MFD as shown in (Fig. 3b), only when fibres are attached to both ends (the most left and right sides), or in the shaded area in Fig. 3b, can they be stretched along the MFD and contribute to the stress response. If only one end of the fibres is stretched (e.g. the unshaded area in Fig. 3b), they will not contribute to the stress response (i.e. the other end is unconstrained). $\alpha _{\text {4f}}(\theta )$ is defined as the ratio between the shaded blue area and the total area of the sample as shown in Fig. 3b, denoting the effective fibre ratio $\varepsilon _\theta$. Similarly, collagen aligned in the CFD may contribute to the stress response depending on the size of the sample, the fibre angle and the experimental set-up. Collagen in the sheet direction is not stretched, which means they do not contribute to the uniaxial test; therefore, for the uniaxial test in Fig. 3b, the effective fibre ratios are

14 $$\begin{aligned}&\quad \alpha _{\text {4f}}(\theta )= \left\{ \begin{array}{rcl} 1-\frac{L_0}{W_0}|\tan (\theta )| &\quad \text{ for } &\quad -\theta _0<\theta<\theta _0, \\ 0 &\quad \text{ for } &\quad \text{ others }, \end{array}\right. \nonumber \\&\quad \alpha _{\text {4n}}(\theta )= \left\{ \begin{array}{rcl} 1-\frac{L_0}{W_0}|\cot (\theta )| &\quad \text{ for } &\quad \frac{\pi }{2}-\theta _0<\theta<\frac{\pi }{2} \, \text {or} \, -\frac{\pi }{2}<\theta <-\frac{\pi }{2}+\theta _0,\\ 0 &\quad \text{ for } &\quad \text{ others }, \end{array}\right. \nonumber \\&\quad \alpha _\text {4s}(\theta ) = 0. \end{aligned}$$

where $W_0$  and $L_0$  are the width and length of the tested sample in the $\mathbf{f}$–$\mathbf{n}$ plane, and $\theta _0 =\arctan \frac{W_0}{L_0}$. Effective ratios for biaxial and simple shear tests can be found in the appendix.

The stress tensor in a myocardium layer ($\det {(\mathbf{F})}=1$) with a specific fibre angle $\theta$ is

15 $$\begin{aligned} \mathbf{P}^\theta = \mathbf{F}\,\frac{\partial \varPsi (\theta )}{\partial \mathbf{F}}\,\mathbf{F}^{-T}-p\mathbf{F}^{-T}. \end{aligned}$$

Because the local fibres in a test sample rotate from $\theta _1$ to $\theta _2$ transmurally (as shown in Fig. 3), the total Cauchy stress tensor for the sample is approximated as:

16 $$\begin{aligned} \mathbf{P}=\frac{1}{\theta _2-\theta _1}\,\int _{\theta _1}^{\theta _2} \mathbf{P}^\theta d\theta . \end{aligned}$$

### Parameter estimation

For Dokos et al. study, all six shear experiments are used for formulating (17). For Sommer et al. study, we fit the strain energy functions using both the biaxial and simple shear tests. All three modes of experimental data from Ahmad et al. study are combined together. Material parameters are estimated using a nonlinear least square minimization function (*fmincon* from MatLab, MathWorks 2017), with the loss function

17 $$\begin{aligned} L({{\varvec{\beta }}})=\sum _{\text {n=1}}^{N}\,[P_n({{\varvec{\beta }}})-P_n^\text {exp}]^2, \end{aligned}$$

where ${{\varvec{\beta }}}$ denotes the set of unknown parameters, *N* is the total number of data points and $P_n^\text {exp}$ are the experimental values. The relative and absolute differences of the area-under-the-curve between the experimental and fitted stress–strain curves ($\text {err}^\text {Relative}$, $\text {err}^\text {Absolute}$) are introduced to quantitatively describe the goodness of fit,

18 $$\begin{aligned} \begin{aligned}&\text {err}^\text {Relative} = \frac{ \int _{\lambda _\text {min}}^{\lambda _\text {max}} |P_n({{\varvec{\beta }}}) - P_n^\text {exp}|\,d\lambda }{\int _{\lambda _\text {min}}^{\lambda _\text {max}} P_n^\text {exp}\,d\lambda }, \\&\text {err}^\text {Absolute} =\int _{\lambda _\text {min}}^{\lambda _\text {max}} |P_i({{\varvec{\beta }}}) - P_n^\text {exp}|\,d\lambda , \end{aligned} \end{aligned}$$

in which $\lambda _\text {min}$ and $\lambda _\text {max}$ are the minimum and maximum stretch or shear, respectively. A value of 0 indicates a perfect fitting.

### Reduced HO models

Some of the invariants may be excluded in the general HO model when applied to human myocardium, whilst still achieving a good agreement with experimental data. For example, Holzapfel and Ogden (2009) reported that after dropping $I_\text {4n},I_\text {8fn}$ and $I_\text {8sn}$ from the general HO model, they could still fit the six shear tests of Dokos et al. (2002) very well; hence, they proposed an 8-parameter HO model (HO2009),

19 $$\begin{aligned} \begin{aligned} \varPsi&=\frac{a}{2b}\exp [b(I_1-3)]+\sum _{i=\text {f,s}}\frac{a_i}{2b_i} \{\exp [b_i( \max (I_{4i}, 1) -1)^2]-1\} \\&\quad +\frac{a_\text {fs}}{2b_\text {fs}}[\exp (b_{\text {fs}}I_\text {8fs}^2)-1]. \end{aligned} \end{aligned}$$

The reason for excluding $I_\text {8fn}$ and $I_\text {8sn}$ is because the two shear responses marked as (nf) and (ns) were not distinguishable based on Dokos et al. data. There lacks, however, a study investigating whether the general HO and HO2009 can fit all other myocardial experiments well, such as human myocardium in Sommer et al. (2015b).

Reducing the general HO model (11) is advantageous, as so many strain invariants and material parameters prevent efficient personalized cardiac simulations. Furthermore, multiple sets of optimal material parameters from limited experimental data can lead to different simulation results for a given boundary-value problem (Ogden et al. 2004). To derive a simplified but competent strain energy function, the AIC analysis (Burnham and Anderson 2003) is employed in this study to reduce the general HO model, which is defined as

20 $$\begin{aligned} \text {AIC}=N\,\ln {\left[ \frac{1}{N}L({{\varvec{\beta }}})\right] }+2K, \end{aligned}$$

where *K* is the number of model parameters. AIC is typically used for model selection by considering both the model complexity and the loss function. The best model is the one with the lowest AIC value. This approach has previously been used by Schmid et al. (2006) to compare five different myocardial strain energy functions. Note the AIC in (20) is negative when the fitting is good. Therefore, for any two different models and the same experimental data, the one with the more negative AIC value suggests a better fitting. Similar AIC values represent comparable models. In this study, we consider various reduced forms of (11). This allows us to drop the terms in (11) that make little change in the AIC value. This way we can select the simplest strain energy function that fits to the test data. To this end, we introduce the AIC ratio:

21 $$\begin{aligned} \eta =\frac{\text {AIC}^\text {model}_\text {reduced}}{\text {AIC}^\text {model}_\text {general}} \end{aligned}$$

where $\eta$ represents the ratio of AIC values of a reduced and the general HO model for the same experimental data.

We aim to simplify the general HO model with a subset of strain invariants ($\{I_\text {4f}$, $I_\text {4s}$, $I_\text {4n}$, $I_\text {8fs}$, $I_\text {8fn}$, $I_\text {8sn}\}$), for effectively characterizing different experimental studies. The steps for reducing the general HO model are

1. Compute the AIC value for the general HO model and $\eta =1$;
2. Compute $\eta$ values for reduced models whilst removing one strain invariant at a time;
3. The invariant associated with the least changed $\eta$ value may be dropped, leading to a reduced HO model,
4. Repeat 2–3 for the remaining set of the strain invariants,
5. If the $\eta$ value is reduced by a predetermined threshold $\epsilon$, stop; otherwise, go to 2.

In this study, we chose $\epsilon$ to be 0.05.

**Fig. 4:**
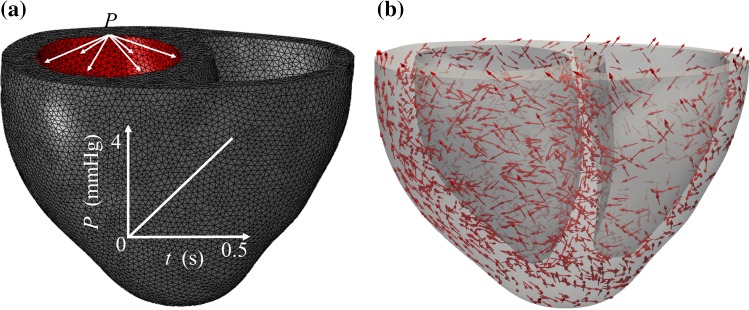
**a** The 3D FE bi-ventricle mesh geometry with a pressure boundary condition applied to the left ventricle inner surface (red surface). The pressure linearly increases from 0 to 4 mmHg in a period of 0.5s. **b** The myofibre distribution in the ventricle wall, which rotates from epicardium to endocardium ($60^o$ to $-60^o$)

We further compare the modelling accuracy between the general and various reduced HO models using a three-dimensional (3D) finite element (FE) bi-ventricular model, which is reconstructed from 3D computed tomography (CT) data. Details of the data acquisition can be found in Ahmad et al. (2018). The 3D CT data are first segmented using Seg3D;^1^ then, the boundary contours are exported into SolidWorks (Dassault Systemes, MA USA) for 3D geometry reconstruction, and then meshed with ICEM (ANSYS, Inc. PA USA). Finally, explicit Abaqus (Dassault Systemes, MA USA) is used for the FE simulation. User subroutines are implemented for different strain energy functions. Diastolic filling in the left ventricle (Fig. 4a) is simulated with layered myofibre rotating from the epicardial to endocardial surface (Fig. 4b), with rotation angles measured from experimental studies using a rule-based approach (Wang et al. 2013).

### Optimal combination of experiments through predictive analysis

Likewise, we can use AIC method to determine the optimal combination of experiments using minimum tests. For a given strain energy function, we firstly fit it to a subset of experimental data with $N_s$ data points and then use it to predict the remaining points ($N-N_s$). We introduce a similar AIC ratio

22 $$\begin{aligned} \delta =\frac{\text {AIC}^\text {exp}_\text {subset}}{\text {AIC}^\text {exp}_\text {all}}. \end{aligned}$$

where $\text {AIC}^\text {exp}_\text {subset}$ and $\text {AIC}^\text {ext}_\text {all}$ are computed using parameters optimized from a subset or all combinations of experimental data, respectively. We do not consider cases when $\delta$ becomes negative. Hence, $\delta$ denotes the AIC change using different combinations of experiments for the same model. We chose the criterion for the best combination to be the minimum group of tests which satisfies $\delta \ge 0.8$. This corresponds to about 5% change of the relative error in (18). The pseudo-code for this analysis is listed in Algorithm 1.

**Algorithm 1** The predictive analysis for determining the optimal combination with minimal tests

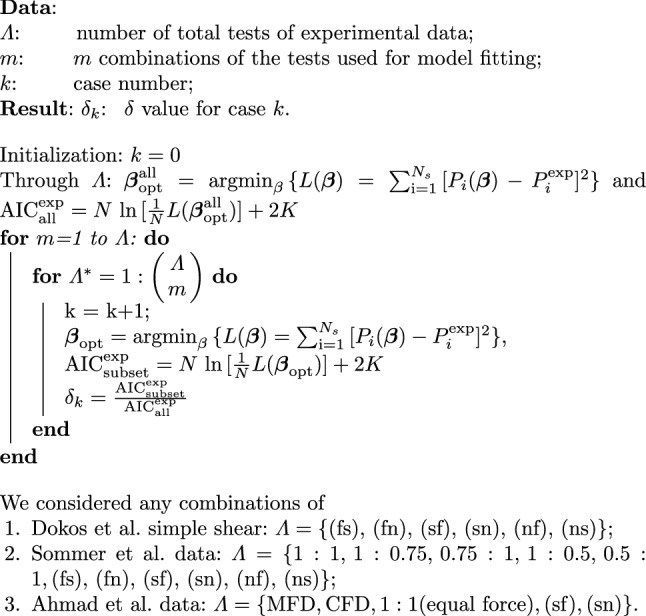



## Results

### The general HO strain energy function

**Fig. 5:**
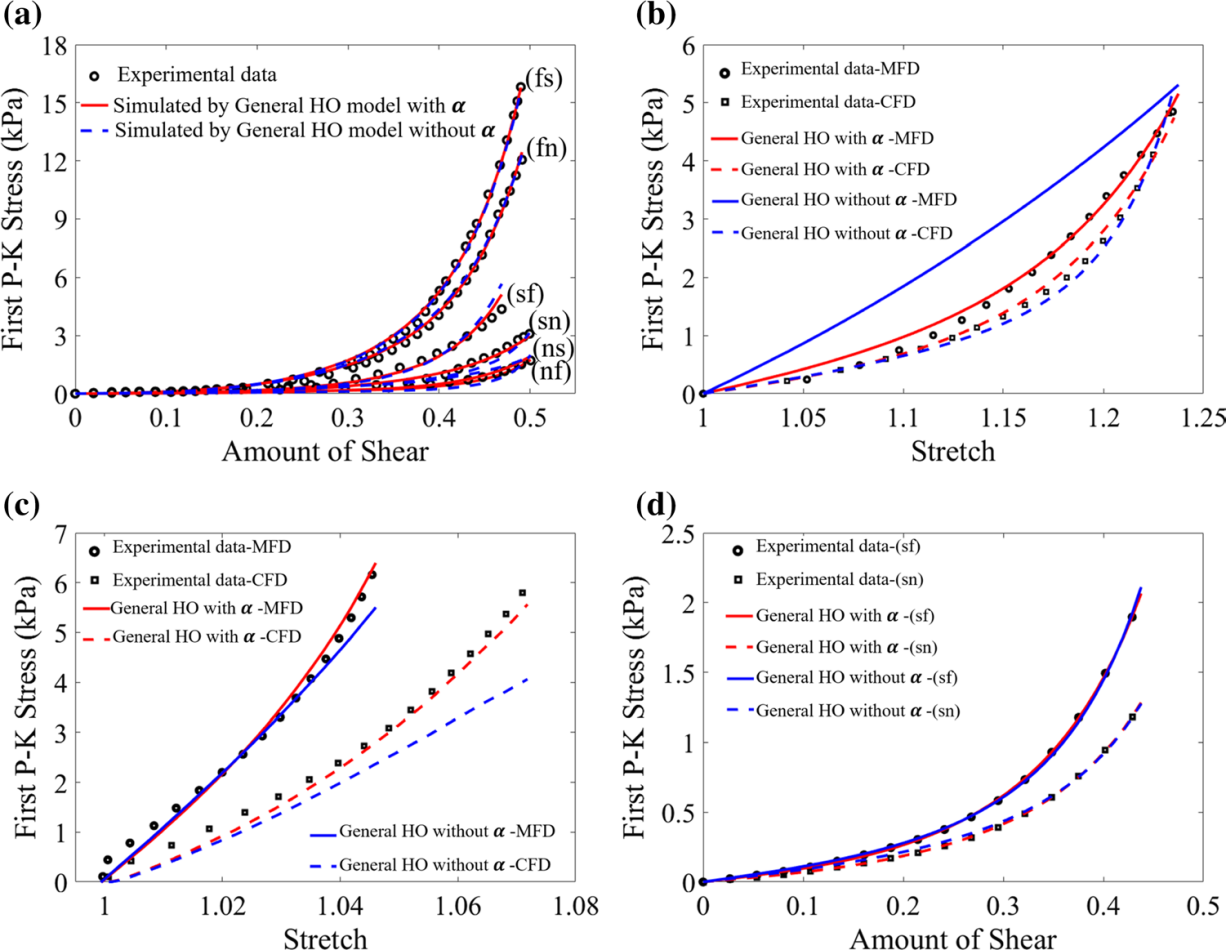
Comparison of the fitting results with and without considering fibre effective ratio ($\alpha$). **a** Fitting the general HO model to Dokos’s data, **b**–**d** the differences in uniaxial, biaxial and simple shear tests in Ahmad’s data

Figure 5a shows the results by fitting the general HO model to the Dokos et al. shear tests. Improved agreement can be found when including the effective fibre ratio (AIC: $-589.3$) compared to without (AIC: $-464.7$), whilst the mean relative error also decreases from 15.9% to 9.3%. When fitting all test data from Ahmad et al. study with the effective fibre ratio, the AIC value is reduced significantly from $-338.5$ to $-1170.3$ (shown in Figs. 5b–d), whilst the relative errors for the uniaxial test along the MFD decrease from 36.06% to 4.25%, and from 26.76% to 6.97%, for the biaxial test along the CFD.

**Fig. 6:**
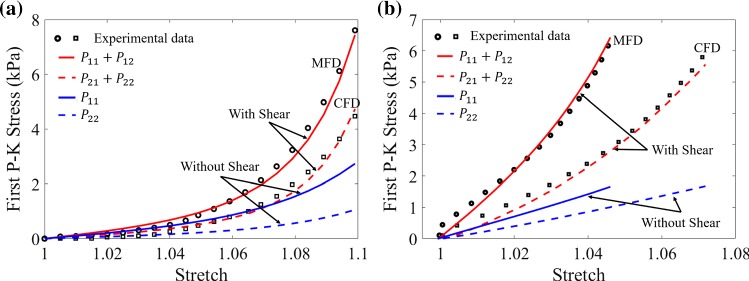
Comparison of the first P–K stress, including shear (red) and not including shear (blue) using same strain energy function. **a** is for Sommer et al. biaxial test and a minimum shear angle of $6^o$ is introduced. Below $6^o$ there is no good fit, above it is not supported by Sommer et al. experiments. **b** is for Ahmad et al. biaxial test, corresponding to Fig. 8e

In Ahmad et al. data, we estimate $k_1=0.18$ and $k_2=0.05$ using markers for the sample angle changes (Fig. 2). In Sommer et al. (2015b), no information on the shear measurements is available. However, we assume the maximum shear angles in both MFD and CFD in Sommer et al. biaxial tests are around $6^\circ$, i.e. $k_1=k_2\approx 0.1$ (Sommer et al. 2015a; Billiar and Sacks 2000), which is necessary for a good fit to their experiments. As for Ahmad et al. and Sommer et al. biaxial tests, the difference with and without shear for the same model in Fig. 6 indicates including the shear component is critical when fitting biaxial experimental tests, since for fibre-reinforced material, it is almost impossible to conduct biaxial tests without inducing shear (Freed et al. 2010).

**Fig. 7:**
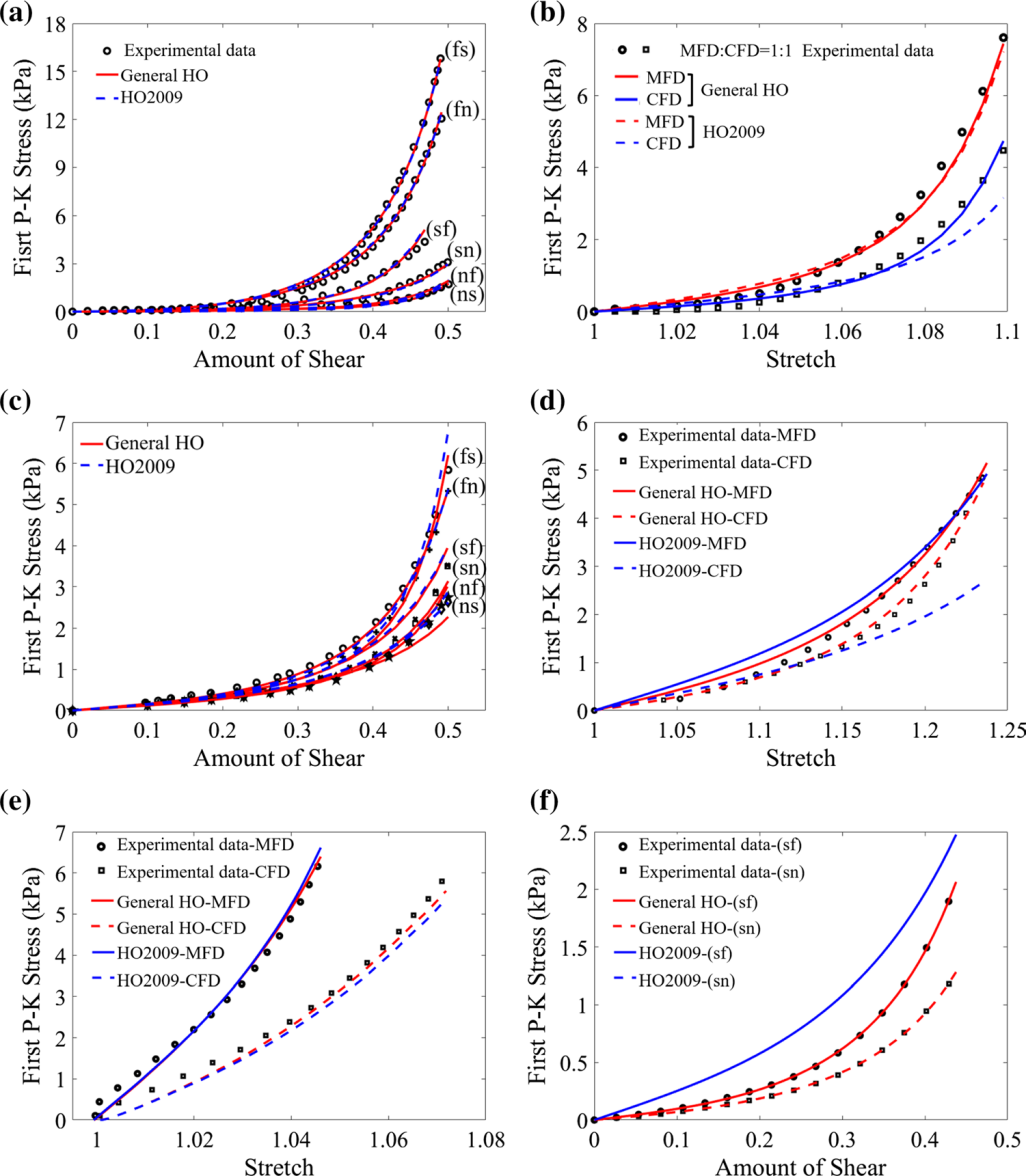
Comparison between descriptive ability of the general HO and the HO2009 models for the three experimental studies. **a** Dokos’s simple shear tests; **b** and **c** Sommer’s biaxial tension and simple shear tests; **d**–**f** Ahmad’s uniaxial, biaxial tension and simple shear tests

Figure 7a demonstrates that both the general HO (AIC: $-589.3$) and HO2009 (AIC: $-559.3$) models can fit Dokos et al. shear test data very well, whilst noticeable differences can be found when fitting the two material models to Sommer et al. data (Fig. 7b, c, where only plotting one set (MFD:CFD=1:1) experimental data, whilst the remaining four sets have similar results and are included in the appendix (Fig. 12). Better agreement is achieved for the general HO model (AIC: $-1102.6$) than the HO2009 model (AIC: $-849.5$). Figure 7d–f shows the results when fitting the two models to Ahmad’s data. Again, much better agreement can be found when using the general HO model (AIC: $-1170.3$) compared to the HO2009 model (AIC: $-423.1$); in particular, the HO2009 model fails to fit the shear test in (Fig. 7f).

### Reduced strain energy functions based on AIC analysis

Although the general HO model can fit the three selected experimental studies very well as shown in Fig. 7, it includes seven invariants with fourteen unknown parameters, which can be extremely challenging to obtain an unique solution when fitting to limited experimental data. A reduced form, such as the HO2009 model, is desirable for constructing personalized models (Gao et al. 2017; Palit et al. 2018; Nikou et al. 2015). However, since HO2009 is derived from fitting the Dokos et al. data only, if such a model fails to describe other experimental data, we need to have strategies in place to derive a better reduced model with test data available for tissues of interests.

**Fig. 8:**
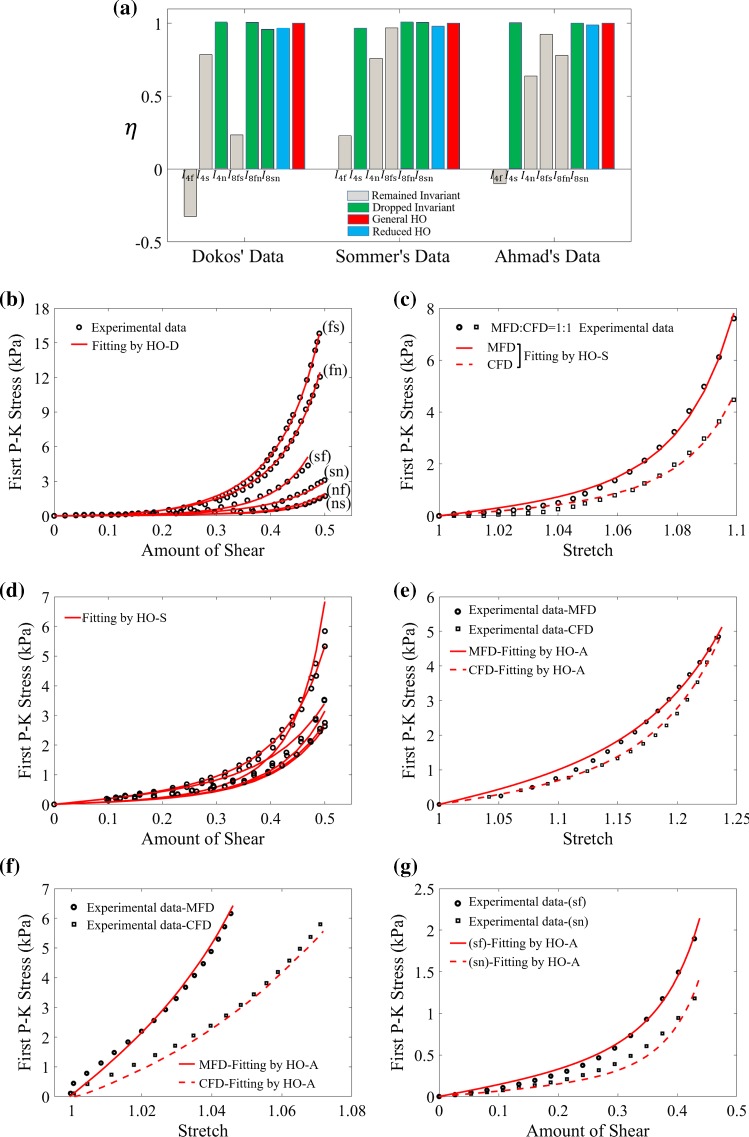
Descriptive capability of reduced HO models. **a** Change of $\eta$ when dropping the terms associated with the invariants for the different three experiments. The fitting results for the HO-D model (**b**), and **c**–**d** the HO-S model and **e**–**g** the HO-A model

In this work, alternative reduced strain energy functions are identified from the general HO model for selected experimental studies based on the AIC analysis. Figure 8a reports $\eta$ values when individually excluding each invariant from the general HO model (11) and fitting to three experimental studies. For Dokos et al. data, $I_\text {4n}$, $I_\text {8fn}$ and $I_\text {8sn}$ have much less contribution to the agreement compared to $I_\text {4f}$, $I_\text {4s}$ and $I_\text {8fs}$, because $\eta$ remains more than 0.95 when dropping these terms. This means $I_\text {4n}$, $I_\text {8fn}$ and $I_\text {8sn}$ can be dropped from (11), and it can now be denoted as HO-D, which actually equates to the HO2009 model (19). In other words, $\eta$ is reduced by 3% when using the HO-D model (HO2009) to replace the general HO model. Similarly, invariants $I_\text {4s}$, $I_\text {8fn}$ and $I_\text {8sn}$ may be excluded from the general HO model when fitting to Sommer et al. biaxial and simple shear data. This gives us a reduced strain energy function for Sommer’s data (HO-S) at a 4% of drop in $\eta$,

23 $$\begin{aligned} \begin{aligned} \varPsi&=\frac{a}{2b}\exp [b(I_1-3)]+\sum _{i=\text {f,n}}\,\frac{a_i}{2b_i} \{\exp [b_i(\text {max}(I_{4i},1)-1)^2]-1\} \\&\quad +\frac{a_\text {fs}}{2b_\text {fs}}[\exp (b_{\text {fs}}I_\text {8fs}^2)-1]. \end{aligned} \end{aligned}$$

Figure 8c shows the fitting results of the HO-S model to various biaxial tests with different stretch ratios, and fitting results to the shear tests are shown in Fig. 8d. Notice, good agreement for Sommer’s biaxial data can only be achieved when a small amount of shear is included. The reduced model for Ahmad et al. data in Fig. 8a, (HO-A), is similarly determined

24 $$\begin{aligned} \begin{aligned} \varPsi&=\frac{a}{2b}\exp [b(I_1-3)]+\sum _{i=\text {f,n}}\,\frac{a_i}{2b_i} \{\exp [b_i(\text {max}(I_{4i},1)-1)^2]-1\} \\&\quad +\sum _{ij=\text {fs,fn}}\,\frac{a_{ij}}{2b_{ij}}[\exp (b_{{ij}}I_{8ij}^2)-1], \end{aligned} \end{aligned}$$

in which $I_\text {4s}$, and $I_\text {sn}$ are excluded from the general HO model, and $\eta$ is only reduced by 0.015. Figure 8e–g shows the fitting results to the uniaxial stretch, biaxial stretch and simple shear tests, respectively. Again, the HO-A model has good descriptive capability for Ahmad et al. experiments. All estimated parameters for the HO-D (HO2009), HO-S and HO-A models and the fitting errors with their corresponding experimental data, can be found in Tables 1 and 2.

**Table 1:** The estimated parameters for the reduced HO models fitting to corresponding experimental studies

		*a* (kPa)	*b*	$a_\text {f}$ (kPa)	$b_\text {f}$	$a_\text {s}$ (kPa)	$b_\text {s}$	$a_\text {n}$ (kPa)	$b_\text {n}$	$a_\text {fs}$ (kPa)	$b_\text {fs}$	$a_\text {fn}$ (kPa)	$b_\text {fn}$	$a_\text {sn}$ (kPa)	$b_\text {sn}$
Dokos et al	HO-D	0.073	15.529	25.992	9.348	4.822	0.001	–	–	0.178	16.740	–	–		–
Sommer et al	HO-S	0.809	7.474	1.911	22.063	–	–	0.227	34.802	0.547	5.691	–	–	–	–
Ahmad et al	HO-A	0.075	18.143	7.067	1.339	–	–	2.745	4.497	1.859	4.066	3.541	8.222	–	–

**Table 2:** Relative and absolute errors for the reduced HO models when fitting to corresponding experimental studies

Experiment	Model		Relative Error ($\%$) and Absolute Error (kPa)																Mean
Dokos et al			(fs)	(fn)	(sf)	(sn)	(nf)	(ns)											
	HO-D	%:	2.96	4.43	12.55	12.10	13.37	16.87											**10.38**
		kPa:	0.12	0.14	0.14	0.11	0.08	0.10											**0.12**
Sommer et al			1:1		1:0.75		0.75:1		1:0.5		0.5:1		(fs)	(fn)	(sf)	(sn)	(nf)	(ns)	
			MFD	CFD	MFD	CFD	MFD	CFD	MFD	CFD	MFD	CFD							
	HO-S	%:	10.12	12.99	13.19	15.83	16.76	9.57	15.29	16.23	31.24	18.22	6.39	15.10	12.34	21.92	5.31	8.54	**14.32**
		kPa:	0.18	0.13	0.16	0.10	0.15	0.06	0.16	0.07	0.14	0.08	0.11	0.23	0.13	0.22	0.05	0.08	**0.13**
Ahmad et al			Uniaxial		Biaxial		Simple shear												
			MFD	CFD	MFD	CFD	(sf)	(sn)											
	HO-A	%:	4.72	4.08	5.95	6.96	7.15	14.61											**7.24**
		kPa:	0.02	0.01	0.04	0.04	0.001	0.005											**0.02**

**Fig. 9:**
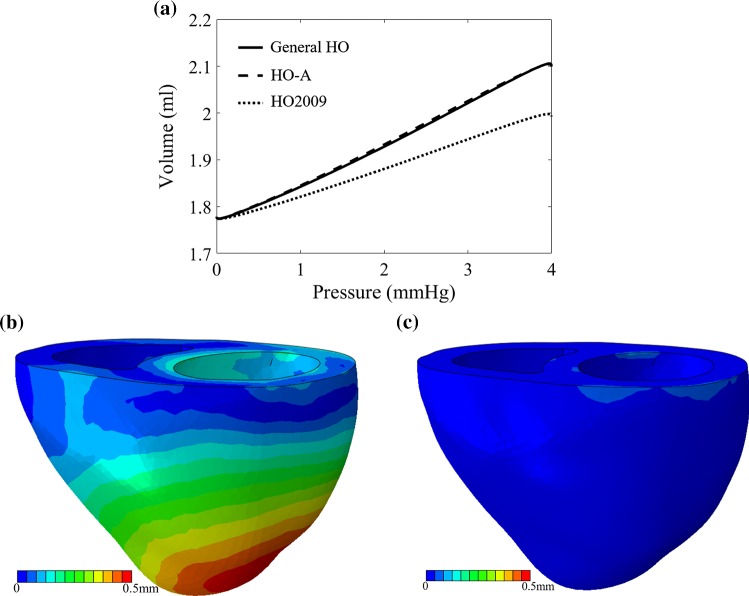
The differences of FE bi-ventricle model using the HO2009, HO-A and general HO models for Ahmad et al. data. **a** The pressure–volume curve in diastolic filling, **b** the displacement differences between the general HO and HO2009 models and **c** the displacement differences between the general HO and HO-A models

Figure 9a describes the left ventricular pressure–volume relationship from the 3D FE bi-ventricle model using the general HO, HO2009 and HO-A models with parameters determined from Ahmad et al. study. Nearly identical pressure–volume relationships can be found between the general HO and HO-A models; however, the ventricle is stiffer when using the HO2009 model even though the parameters are determined using the same experimental data. This appears to indicate that the HO2009 model cannot effectively characterize Ahmad et al. myocardial samples. We further compare the displacement differences among different material models based on Ahmad et al. data. The displacement differences between the general HO and HO-A models are nearly negligible (Fig. 9c), but large discrepancies exist for the HO2009 model (Fig. 9b).

### Optimal combination of experimental tests

To find the optimal combination of tissue tests, we use reduced HO models and a random initialization strategy to get the average value of $\delta$, $avg(\delta )$, and its corresponding standard deviation, *std*.

**Fig. 10:**
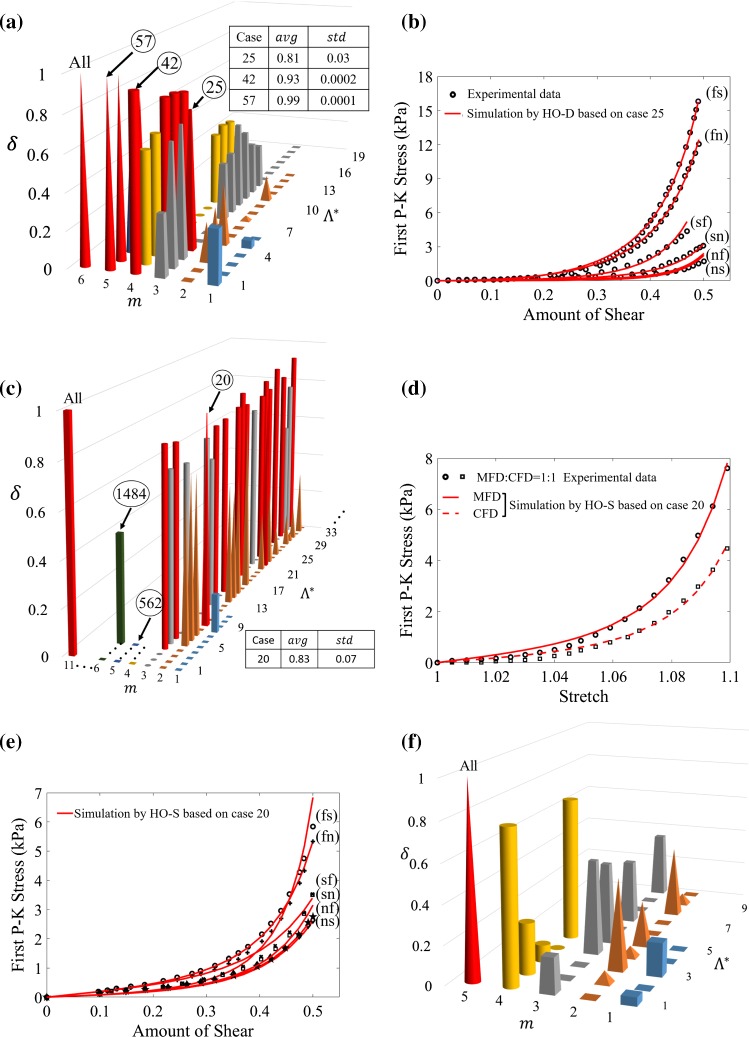
$\delta$ values that are computed according to Algorithm 1, where the cases whose average (*avg*) $\delta \ge 0.8$ are marked in red. **a** In Dokos et al. experiments, case 25 ((fs) + (fn) + (ns)) is the optimal case which has few tests whilst meeting the criterion, **b** the corresponding fitting curves using case 25. In Sommer et al. experiments, case 20 ((1:1) + (nf)) is the optimal case as shown in **c**, and the corresponding fitting curves are shown in **d** and **e**. **f** is for Ahmad et al. experiments, case ALL is the only one which satisfies the criterion. The other cases are corresponding to certain combinations to be discussed in the text

**Combinations from Dokos et al. data** As shown in Fig. 10a, in additional to all tests, case 25 ($\text {(fs)}+\text {(fn)}+\text {(ns)}$), case 42 ($\text {(fs)}+\text {(fn)}+\text {(sf)}+\text {(sn)}$), case 57 ($\text {(fs)}+\text {(fn)}+\text {(sf)}+\text {(sn)}+\text {(nf)}$) meet the criterion of $avg(\delta ) \ge 0.8$. Clearly, case 25 is the optimal combination.

**Combinations from Sommer et al. data** Figure 10c displays partial $avg(\delta )$ values of Sommer et al. AIC analysis when combining different biaxial and simple shear test data using the HO-S model; for clarity, only group 1, 2, 3, 5 and 6 are shown. The best combination is case 20 ((1:1)+(nf)). In particular, case 562 ((1:1)+(1:0.75)+(0.75:1)+(1:0.5)+(0.5:1)) is the combination of all biaxial data and has negative $\delta$ value, suggesting using biaxial data only cannot predict the simple shear responses. Likewise, using simple shear tests only, case 1484 ($\text {(fs)}+\text {(fn)}+\text {(sf)}+\text {(sn)}+\text {(nf)}+\text {(ns)}$) is unable to predict biaxial data. Therefore, both biaxial and simple shear test data are needed when characterizing myocardial properties. This agrees with the observation by Holzapfel and Ogden (2009). Figure 10d, e shows the stress of biaxial tests and simple shear tests with parameters determined from stress responses in (1:1)+(nf).

**Combinations from Ahmad et al. data** In Fig. 10f, apart from all tests, none of the other combinations meet $\delta \ge 0.8$ in Ahmad et al. study. The fitting curves using all the tests are already shown in Fig. 8e, g, f.

## Discussion

This study focuses on a rational reduction of the general HO model for the myocardial tissue responses. Three different myocardial experiments are selected, including Dokos et al. study on porcine myocardium over a decade ago (Dokos et al. 2002), Sommer et al. study on human myocardium published several years ago (Sommer et al. 2015b), and the very recent experimental data from Ahmad et al. (2018) on neonatal porcine myocardium (Ahmad et al. 2018). To our best knowledge, these are the most comprehensive myocardial mechanical experiments. Dokos et al. (2002) is the first presenting simple shear tests to characterize the direction-dependent myocardial mechanical property, which has driven new developments in strain energy function and led to the extensive use of the HO2009 model (Holzapfel and Ogden 2009). Sommer et al. (2015b) included biaxial and simple shear tests, with both needed for characterizing an orthogonal hyperelastic material (Holzapfel and Ogden 2009). We show, for the first time, that the general HO model is very good as describing stress responses from different deformation types as shown in Fig. 7.

A number of studies have used the HO-based strain energy functions (mostly HO2009 model) to construct personalized biomechanical models (Gao et al. 2017; Asner et al. 2016; Baillargeon et al. 2014). The widely successful application of the HO-type models suggests it is good for characterizing myocardial mechanical properties and provides the natural starting point to optimize the general HO model for specific tissue types, aiming to achieve the least terms and yet retaining sufficient descriptive and predictive capability. However, it has been recognized that the HO2009 model has its limitations (Fig. 7). This is because their model reduction is based on Dokos et al. simple shear data only, which did not include all responses of the myocardial tissues.

In the past several decades, efforts have been made to develop a strain energy function with fewest terms, whilst accurately describing the test data and predicting the dynamics (Zhang et al. 2019). A simplified but competent material model not only reduces computational cost, but is also easy to implement and personalize from limited test data. In this study, the AIC analysis is employed to systematically reduce the general HO model, whilst maintaining good descriptive and predictive capabilities. An invariant is excluded from the general HO model if it causes only a small change in the resultant AIC value. For instance, Fig. 8a suggests that $I_{4\text {n}}$, $I_{8\text {fn}}$ and $I_{8\text {sn}}$ could be excluded when fitting to the Dokos et al. data, which is the same formulation as the HO2009 model. Other approaches can also be used for model reduction and selection such as parameter sensitivity analysis, by setting those insensitive parameters to constant values or zero (Snowden et al. 2017).

Interestingly, the reduced HO models are different for the selected experimental studies. Presumably, this is because these tests were for different species and ages; Dokos et al. (2002) used adult porcine myocardium, Ahmad et al. (2018) used the neonatal porcine myocardium, and Sommer et al. (2015b) worked on human myocardium. When fitting to the biaxial tests only from Sommer et al. data, the general HO model can be simplified to a reduced form consisting of only $I_1$ and $I_{4\text {f}}$, similar to the findings reported in Holzapfel and Ogden (2009). This is because in the biaxial tests, collagen fibres are only stretched in fibre-normal plane, but not in the sheet direction, thus $\max ({I_{4\text {s}}}, 1) = 1$ and $I_{8\text {fs}} = 0$. When fitting to the biaxial and simple shear tests together, the term with $I_{4\text {n}}$ needs to be included, which is different from the reduced formulation when fitting only to Dokos et al. data. One reason is that the shear responses along (fs) and (fn) are closer to each other in Sommer et al. human myocardium, than in Dokos et al. porcine myocardium. This is similar to shear responses along (sf) and (sn), and along (nf) and (ns), which suggests there may be a difference in passive myocardial properties between human and porcine myocardium. The reduced HO model from Ahmad et al. data needs to incorporate $I_{8\text {fn}}$, which might be explained by: (1) the asymmetric fibre structure in relation to the stretching axis; and (2) limited test data with only 2 shear responses, 2 biaxial tests and 2 uniaxial tests. There is, however, no conclusion as to the number of tests required with different deformation types to fully characterize myocardium.

The AIC analysis can also be used to choose the best combination of experiments. As shown in Fig. 10, different combinations of test data affect the prediction accuracy. Specifically, within the shear responses (Fig. 10a), the groups containing (fs) and (fn) always have better predictive capability than other groups. One reason is that the shear responses along (fs) and (fn) are much stiffer than other directions in both Dokos et al. and Sommer et al. data. For the biaxial test, most combinations have good predictive capability, which suggests that not all the biaxial tests in Sommer et al. data are needed to fit the general HO model or the HO-S model. For instance, one stretching ratio with 1(MFD):0.75(CFD) from Sommer et al. biaxial tests has good predictions for other stretching ratios. But if the stretch ratio is largely non-equal, such as 1(MFD):0.5(CFD) or 0.5(MFD):1(CFD), the prediction is poor (see Fig. 13 in Appendix), partially because the material response with lower stretch ratios is still within the toe regime with non-stretched collagen fibres (Cheng et al. 2018; Lanir 1979). Prediction between different deformation types is poor, as shown in Fig. 10b, using biaxial tests only (case 563) and simple shear only (case 1484). This might be because one experiment type is inadequate to capture the nonlinearity and anisotropy of myocardium. Ahmad et al. (2018) included simple shear, biaxial and uniaxial tests, which allows investigation of uniaxial data in characterizing myocardium property. However, even with Ahmad et al. data, the predictions of uniaxial tests using the two biaxial and simple shear tests (case 25) are poor. As discussed in Holzapfel and Ogden (2009), biaxial tests are insufficient for characterizing a hyperelastic anisotropic material. When using stress responses from both the simple shear and biaxial tests, the least test data for the HO-S model with good prediction are one shear test along (nf), together with a biaxial test 1(MFD):1(CFD). Our results presented here suggest uniaxial tests are still needed for an experiment like Ahmad et al. study, whilst further studies may be needed for experiments like Sommer et al. study using uniaxial tests.

**Fig. 11:**
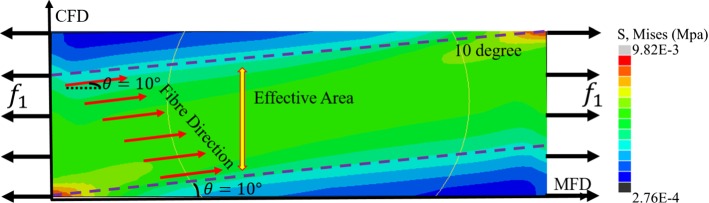
Stress distribution when fibre direction is $10^\circ$ in uniaxial tensile along MFD as shown in Fig. 3. The green area enclosed by the two dashed lines is the effective area with higher stress, whilst the blue area (the right bottom and left upper corners ) is the ineffective area with much lower stress

**Fig. 12:**
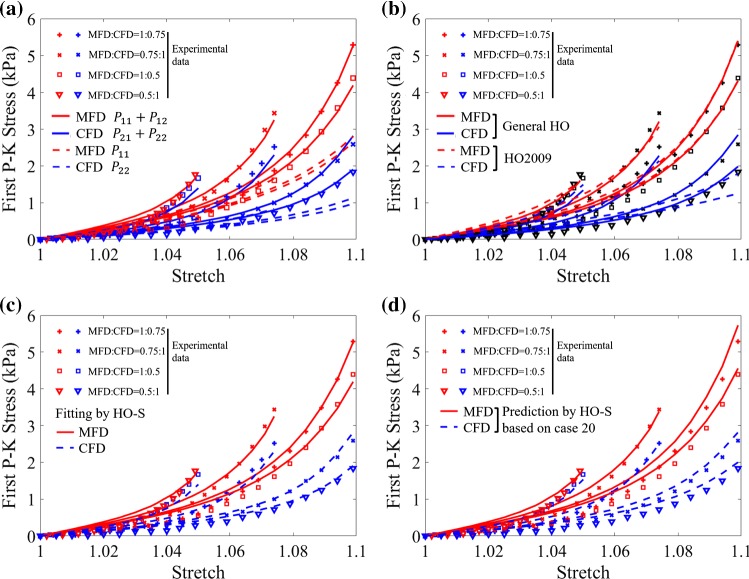
The rest four loading protocols [(1:0.75), (0.75:1), (1:0.5) and (0.5:1)] for Sommer et al. biaxial tests. **a** Comparison of the first P–K stress including shear (solid lines) or not (dash lines) as in Fig. 6a. **b** The comparison between the general HO model and HO2009 model in Fig. 7b. **c** Fitting results using the reduced HO model (HO-S) in Fig. 8c. **d** The simulated results according to optimized combinations (1:1)+(nf) in Fig. 10e

**Fig. 13:**
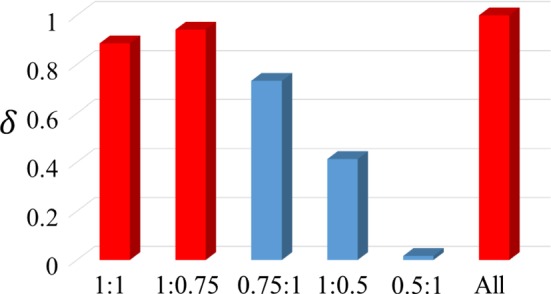
Only using biaxial tests in Sommer et al. data, we compute $\delta$ by fitting the HO-S model to one individual stretch ratio and predicting the remained experimental data from other stretch ratios. Only (1:1) and (1:0.75) meet $\delta \ge 0.8$, whilst (1:0.5) and (0.5:1) have $\delta \le 0.4$

In general, the stiffness aligned to the collagen fibre direction is much greater than the extracellular matrix, which is considered homogeneous and isotropic. Many studies have demonstrated the importance of excluding compressed fibres which cannot bear load (Zhuan et al. 2018; Holzapfel and Ogden 2017). Here we use a simpler approach, effective fibre ratios, to consider this effect. Because of the gradual fibre rotation transmurally, we assume the collagen fibres will experience the same deformation as the extracellular matrix only when both ends are stretched. A simplified FEM model based on Fig. 3b is simulated under uniaxial stretch along the MFD (Fig. 11), showing that the stress is much higher in the effective fibre area. The inclusion of the effective fibre ratio is also supported by Fig. 5, where the goodness of fit for the general HO model is much better than without it. The effective fibre ratio is a geometrical effect and depends on the sample size, loading direction and the local collagen fibre structures. It does not affect the fit to biaxial tests since the in-plane collagen fibres will be physically stretched at both ends, but will affect the fit to the uniaxial and simple shear tests.

This study also demonstrates that biaxial stretch of myocardium cannot be free of shear. The shear-free scenario is only possible if fibres are strictly aligned in both stretching directions and without cross-fibre effects. Both are not true in mycardium tissue tests. The assumption of no shear in the model leads to the poor outcome of predicting biaxial test data from simple shear tests, even if the general HO model is used. Indeed, we show that assuming shear-free behaviour in Sommer et al. biaxial testing produced relatively poor goodness of fit for both the general HO and HO-S models; however, this is significantly improved when including a small shear component as per biaxial tests of fibre-reinforced anisotropic material (Sommer et al. 2015a; Billiar and Sacks 2000)(Figs. 6a). As the shear components in the biaxial tests are not reported by Sommer et al. (2015b), the maximum shear angles are assumed to be the same along the CFD and MFD, respectively, at around $6^o$. In Ahmad et al. data, the shear components in the biaxial tests are estimated, with the results presented here (Fig. 6b) suggesting that measuring of shear components in biaxial testing is necessary for myocardium and potentially, other anisotropic materials.

To determine the variability of material parameters when fitting the various HO models to the experimental data, a random initialization strategy is used with 100 samples drawn from predefined parameter ranges. Estimated parameters from different guesses are summarized in Table 3. In general, all estimated parameters for reduced HO models have small standard deviations compared to the average values, and also less than the standard deviations from the general HO model, suggesting a better determinability for reduced HO models. The large standard deviations in the general HO model are expected because it has more parameters. It is also noted when fitting to Dokos et al. data that some parameters lie in the lower bounds such as $b_\text {s}$ in the HO-D model. This may be partially explained by the limited experimental data, which cannot capture some directional stress responses, or due to the interdependence of material parameters (Gao et al. 2015). Since no studies exist on the quantity of experimental data required to fully capture myocardial mechanical properties, we limit this study to three experimental studies in our AIC analysis.

**Table 3:** The average value *avg* and standard deviation *std* of optimized parameters from 100 random generated initial starts in interval (0.001, 50)

Experiment	Model		Parameters													
			*a* (kPa)	*b*	$a_\text {f}$ (kPa)	$b_\text {f}$	$a_\text {s}$ (kPa)	$b_\text {s}$	$a_\text {n}$ (kPa)	$b_\text {n}$	$a_\text {fs}$ (kPa)	$b_\text {fs}$	$a_\text {fn}$ (kPa)	$b_\text {fn}$	$a_\text {sn}$ (kPa)	$b_\text {sn}$
Dokos et al	HO-D	*avg*	0.073	15.517	26.040	9.333	4.869	0.001	–	–	0.170	16.955	–	–	–	–
		*std*	4.0E–3	2.3E–1	1.1E–1	4.3E–2	4.7E–2	5.6E–5	–	–	5.0E–3	1.2E–1	–	–	–	–
	General HO	*avg*	0.019	8.576	25.790	9.668	4.281	0.010	0.001	0.868	0.250	16.037	0.025	13.826	0.252	8.773
		*std*	1.6E–2	7.7E+0	3.4E–2	1.5E–2	3.0E–2	3.8E–2	8.3E–4	6.5E–1	1.8E–2	1.8E–1	2.0E–2	5.7E+0	1.0E–1	4.7E+0
Sommer et al	HO-S	*avg*	0.809	7.474	1.911	22.063	–	–	0.227	34.802	0.547	5.691	–	–	–	–
		*std*	9.5E–4	4.9E–3	1.2E–3	4.8E–3	–	–	1.3E–3	2.2E–2	8.8E–4	1.8E–2	–	–	–	–
	General HO	*avg*	0.180	9.762	2.204	21.597	0.098	49.878	0.508	27.719	1.291	5.295	1.345	2.017	0.947	4.514
		*std*	4.4E–3	7.8E–3	5.1E–3	1.3E–2	1.8E–2	2.6E–1	3.3E–3	1.7E–2	4.9E–3	2.2E–2	1.6E–2	7.2E–1	2.2E–3	5.2E–1
Ahmad et al	HO-A	*avg*	0.075	18.143	7.067	1.339	–	–	2.745	4.497	1.859	4.066	3.541	8.222	–	–
		*std*	2.0E–4	1.6E–2	6.4E–4	9.1E–4	–	–	2.7E–3	6.2E–3	1.1E–3	3.4E–3	2.0E–3	7.2E–3	–	–
	General HO	*avg*	0.005	0.484	7.212	1.25	2.244	13.414	3.223	3.747	1.069	8.961	3.344	11.016	0.421	5.773
		*std*	3.5E–18	6.5E–19	9.0E–4	1.3E–3	3.0E–2	8.4E–1	4.8E–3	9.8E–3	7.7E–4	4.0E–2	2.6E–3	9.5E–3	5.5E–4	1.1E–1

Many other constitutive models exist such as the “pole-zero” model (Nash and Hunter 2000), various Fung-type models (Costa et al. 2001; Guccione et al. 1991) and the constitutive framework with minimized cross-term covariance proposed by Criscione et al. (2002). The AIC analysis can be readily applied to select different types of material models. For instance, we can compare the HO-D model and the Feng-type Guccione’s model (Guccione et al. 1991) with Dokos et al. shear data. We find that better fitting results can be achieved using the HO-D model, which has a much lower AIC value ($-559.3$) than the value from the Guccione’s model ($-65.8$). This is because the Guccione’s model is a transversely isotropic material model, but myocardium is known to be orthotropic.

## Conclusion

This study describes an AIC-based constitutive model reduction for myocardium. We make use of three different myocardial mechanical studies, including uniaxial, biaxial and simple shear tests. We propose three different reduced HO models based on the congressing myocardial tissue studies, with all models retaining similar descriptive and predictive capabilities as the general HO model. We demonstrate the importance of accounting for the shear in the biaxial experiments, as without shear, it is not possible to describe the biaxial experiments reliably. We further demonstrate that it is necessary to consider through thickness fibre rotations in the sample, which is done by introducing the effective fibre ratio when fitting material models to the uniaxial and simple shear myocardial experiments. Finally, we use the AIC analysis to identify the best combinations of tissue tests, and our results show that the minimum one shear response (nf) and one biaxial test with stretch ratio 1(MFD): 1(CFD) are required to capture human myocardial mechanical property in Sommer et al. study. The different reduced material models for the three experimental studies indicate that the least terms required to achieve a competent material model may depend on species, ages and pathologies. Therefore, a combined experimental and modelling approach is important in selecting an appropriate material model for predictive biomechanical models in personalized medicine.

## Appendix

### Effective ratio for biaxial and simple shear

In biaxial tests, because the four sides are stretched along two directions simultaneously in the fibre-normal plane , thus

$$\begin{aligned} \alpha _{\text {4f}}(\theta )=\alpha _{\text {4n}}(\theta )=1, \end{aligned}$$

and $\alpha _{4\text {s}}(\theta )=0$ because the fibre in $\mathbf{s}$ direction is compressed.

There are six different shear modes; fibre effective ratio will be different in every shear mode. If assuming the fibre rotation is from $-\frac{\pi }{4}$ to $\frac{\pi }{4}$ and the specimen is a cube, then we have

$$\begin{array}{*{20}l} {{\text{(fs)}}:\quad \alpha _{{{\text{4f}}}} (\theta ) = \left\{ {\begin{array}{*{20}c} {1 - |\tan (\theta )|} & {{\text{ for }}} & { - \frac{\pi }{4} < \theta < \frac{\pi }{4}} \\ \end{array} } \right.} \hfill & {\alpha _{{{\text{4s}}}} (\theta ) = \alpha _{{{\text{4n}}}} (\theta ) = 0,} \hfill \\ {{\text{(fn)}}:\quad \alpha _{{{\text{4f}}}} (\theta ) = \left\{ {\begin{array}{*{20}c} {1 - |\tan (\theta )|} & {{\text{ for }}} & {0 \le \theta < \frac{\pi }{4}} \\ 0 & {{\text{ for }}} & { - \frac{\pi }{4} < \theta < 0} \\ \end{array} } \right.} \hfill & {\alpha _{{{\text{4s}}}} (\theta ) = \alpha _{{{\text{4n}}}} (\theta ) = 0,} \hfill \\ {{\text{(sf)}}:\quad \alpha _{{{\text{4s}}}} (\theta ) = \left\{ {\begin{array}{*{20}c} 1 & {{\text{ for }}} & { - \frac{\pi }{4} \le \theta < \frac{\pi }{4}} \\ \end{array} } \right.} \hfill & {\alpha _{{{\text{4f}}}} (\theta ) = \alpha _{{{\text{4n}}}} (\theta ) = 0,} \hfill \\ {{\text{(sn)}}:\quad \alpha _{{{\text{4s}}}} (\theta ) = \left\{ {\begin{array}{*{20}c} 1 & {{\text{ for }}} & { - \frac{\pi }{4} \le \theta < \frac{\pi }{4}} \\ \end{array} } \right.} \hfill & {\alpha _{{{\text{4f}}}} (\theta ) = \alpha _{{{\text{4n}}}} (\theta ) = 0,} \hfill \\ {{\text{(nf)}}:\quad \alpha _{{{\text{4n}}}} (\theta ) = \left\{ {\begin{array}{*{20}c} 0 & {{\text{ for }}} & {0 \le \theta < \frac{\pi }{4}} \\ {1 - |\tan (\theta )|} & {{\text{ for }}} & { - \frac{\pi }{4} < \theta < 0} \\ \end{array} } \right.} \hfill & {\alpha _{{{\text{4f}}}} (\theta ) = \alpha _{{{\text{4s}}}} (\theta ) = 0,} \hfill \\ {{\text{(ns)}}:\quad \alpha _{{{\text{4n}}}} (\theta ) = \left\{ {\begin{array}{*{20}c} {1 - |\tan (\theta )|} & {{\text{ for }}} & { - \frac{\pi }{4} < \theta < \frac{\pi }{4}} \\ \end{array} } \right.} \hfill & {\alpha _{{{\text{4f}}}} (\theta ) = \alpha _{{{\text{4s}}}} (\theta ) = 0.} \hfill \\ \end{array}$$

### Rest fitting results in Sommer et al. biaxial test

See Figs. 12 and 13.

## Footnotes

1
http://www.sci.utah.edu/cibc-software/seg3d.html
